# Serum Cadmium Levels in Pancreatic Cancer Patients from the East Nile Delta Region of Egypt

**DOI:** 10.1289/ehp.8035

**Published:** 2005-08-25

**Authors:** Alison M. Kriegel, Amr S. Soliman, Qing Zhang, Nabih El-Ghawalby, Farouk Ezzat, Ahmed Soultan, Mohamed Abdel-Wahab, Omar Fathy, Gamal Ebidi, Nadia Bassiouni, Stanley R. Hamilton, James L. Abbruzzese, Michelle R. Lacey, Diane A. Blake

**Affiliations:** 1 Department of Biochemistry, Tulane University Health Sciences Center, New Orleans, Louisiana, USA; 2 Department of Epidemiology, University of Michigan School of Public Health, Ann Arbor, Michigan, USA; 3 Department of Epidemiology, University of Texas M.D. Anderson Cancer Center, Houston, Texas, USA; 4 Gastrointestinal Surgery Center, Mansoura University, Mansoura, Egypt; 5 Division of Pathology and Laboratory Medicine, and; 6 Department of Gastrointestinal Medical Oncology, University of Texas M.D. Anderson Cancer Center, Houston, Texas, USA; 7 Department of Mathematics, Tulane University, New Orleans, Louisiana, USA

**Keywords:** cadmium, East Nile Delta region, environmental exposure, immunoassays, occupational exposure, pancreatic cancer, pollution

## Abstract

The northeast Nile Delta region exhibits a high incidence of early-onset pancreatic cancer. It is well documented that this region has one of the highest levels of pollution in Egypt. Epidemiologic studies have suggested that cadmium, a prevalent pollutant in the northeast Nile Delta region, plays a role in the development of pancreatic cancer.

Objective: We aimed to assess serum cadmium levels as markers of exposure in pancreatic cancer patients and noncancer comparison subjects from the same region in Egypt.

Design and Participants: We assessed serum cadmium levels of 31 newly diagnosed pancreatic cancer patients and 52 hospital comparison subjects from Mansoura, Egypt.

Evaluation/Measurements: Serum cadmium levels were measured using a novel immunoassay procedure.

Results: We found a significant difference between the mean serum cadmium levels in patients versus comparison subjects (mean ± SD, 11.1 ± 7.7 ng/mL vs. 7.1 ± 5.0 ng/mL, respectively; *p* = 0.012) but not in age, sex, residence, occupation, or smoking status. The odds ratio (OR) for pancreatic cancer risk was significant for serum cadmium level [OR = 1.12; 95% confidence interval (CI), 1.04–1.23; *p* = 0.0089] and farming (OR = 3.25; 95% CI, 1.03–11.64; *p* = 0.0475) but not for age, sex, residence, or smoking status.

Conclusions: The results from this pilot study suggest that pancreatic cancer in the East Nile Delta region is significantly associated with high levels of serum cadmium and farming.

Relevance to Clinical Practice/Public Health: Future studies should further investigate the etiologic relationship between cadmium exposure and pancreatic carcinogenesis in cadmium-exposed populations.

Pancreatic cancer is one of the most deadly forms of cancer. Although it is the fourth leading cause of cancer deaths in the United States, it accounts for only 2% of all newly diagnosed cancers each year. Five-year survival rates for patients diagnosed with pancreatic cancer in the United States average only 4.4% (American Cancer Society 2005). In developing countries, pancreatic cancer appears to be extremely rare. Studies in Egypt, Algeria, and Iraq suggest a low incidence of the disease in the Middle East (Al-Bahrani et al. 1982; Mokhtar 1991; Parkin 1986; Parkin et al. 1997; Sherif and Ibrahim 1987). Risk factors for this disease have generally been grouped into four categories: *a*) cigarette smoking (Doll et al. 1994; Howe et al. 1991); *b*) chronic pancreatitis (largely from alcohol consumption) (Lowenfels et al. 1993) and genetic predisposition (Brand and Lynch 2000; Flanders and Foulkes 1996; Goggins et al. 1996; Lowenfels et al. 1997); *c*) diabetes mellitus and macro- or micronutrients (Bueno de Mesquita et al. 1992; Everhart and Wright 1995); and *d*) occupational and environmental contamination from exposure to pesticides and fertilizers (Anderson et al. 1996; Fontham and Correa 1989; Hoppin et al. 2000), manufacturing paints and pigments (Norell et al. 1986; Raymond and Bouchardy 1990; Sheffet et al. 1982), metalworking (Mallin et al. 1986; Maruchi et al. 1979; Park and Mirer 1996; Rotimi et al. 1993; Silverstein et al. 1988; Sparks and Wegman 1980), and soldering (Ji et al. 1999).

The incidence of pancreatic cancer in Egypt has been previously studied with particular attention to Dakahlia Province (Soliman et al. 2002). This primarily rural province is the largest of all the provinces in the East Nile Delta region. The population in this region exhibits an unusually high rate of early-onset pancreatic cancer. Although most cases in the United States occur in patients older than 65 years of age (Lowenfels and Maisonneuve 1999), the incidence rates for patients younger than 65 years in the East Nile Delta region were more than twice as high as those observed in Americans in the same age group and significantly higher than those seen in other parts of Egypt (Soliman et al. 2002). The reason for the incidence of early-onset pancreatic cancer in the East Nile Delta region is unclear. There is currently no good evidence that the first three risk factors named above would preferentially affect residents of the East Nile Delta region relative to the rest of Egypt. It is, however, well documented that this region has one of the highest levels of pollution in Egypt.

Nile River water is seriously contaminated with heavy metals, pesticides, and hydrocarbons as a result of increasing discharge of untreated industrial wastes and agricultural irrigation wastewater (Badawy et al. 1995). Several reports on Dakahlia Province show high levels of heavy metal and organocholorine pesticides in the soil and water in this region (Abdel-Haleem et al. 2001; Dekov et al. 1997; Reinhardt et al. 2001; Siegel et al. 1994). High concentrations of heavy metals, including cadmium, are among the pollutants in the water. Plants and fish grown in this water are also contaminated with heavy metals (Abdel-Sabour 2001; Abou-Arab and Abou Donia 2000), which can in turn accumulate in humans and animals that feed on these contaminated foods (Osfor et al. 1998). The serum cadmium levels of residents of Dakahlia Province are almost 10-fold higher than those of residents from cadmium-polluted areas in Cairo and 32 times higher than reference levels for healthy populations in the United States (Hossny et al. 2001; Osfor et al. 1998; Soliman et al. 2002). Cadmium is a known human carcinogen (Boffetta 1993) and has recently been implicated as a cause of pancreatic cancer (Schwartz and Reis 2000). Two main risk factors for pancreatic cancer, age and cigarette smoking, are also associated with cadmium exposure. Cadmium accumulates in the body over time because there are no specific mechanisms for its removal. The half-life of this metal in the body ranges from 10 to 30 years, with an average of 15 years (Jin et al. 1998). In addition, cigarette smoking is a significant source of cadmium. One cigarette contains 1–2 μg cadmium (Goyer 1996), and inhaled cadmium is absorbed much more efficiently than is ingested cadmium (Friberg 1984). Measurement of cadmium in the pancreas of autopsy patients showed significantly higher levels in smokers than in nonsmokers (Elinder et al. 1976). Urinary cadmium levels, commonly used as an indication of life-long exposure, are also significantly higher in smokers versus nonsmokers (Schwartz et al. 2003).

In this study, we assessed serum cadmium levels of newly diagnosed histologically confirmed pancreatic cancer patients with hospital comparison subjects from the same region in Egypt, using a novel immunoassay procedure. We have also examined the contributions of age, residence, smoking status, and profession to overall risk for pancreatic cancer. Although the sample size in this study is small, it is our hope that these initial data will act as a springboard for larger, more in-depth studies that will analyze the relationship between cadmium and pancreatic cancer in a more detailed fashion.

## Materials and Methods

### Materials.

The 2A81G5 monoclonal antibody, which recognizes Cd(II)–EDTA complexes, was prepared from a hybridoma generated in the Blake laboratory (Blake et al. 1996). A Cd(II)–EDTA–horseradish peroxidase (HRP) conjugate was prepared as described by Darwish and Blake (2002, 2001). We purchased a pooled human serum sample from Intergen (Milford, MA, USA); this serum sample was analyzed by graphite-furnace atomic absorption spectroscopy (AAS) and shown to be free of endogenous cadmium (data not shown). ELISA high-binding 96-well plates were a product of Corning-Costar (Cambridge, MA, USA). We obtained AAS standard cadmium (1,000 mg/L in 2% nitric acid) from Perkin-Elmer (Norwalk, CT, USA).

### Patient selection.

Between September 2001 and February 2002, 31 newly diagnosed patients with adenocarcinoma of the pancreas from the Gastrointestinal Surgery Center of Mansoura University, Dakahlia Province in Egypt were recruited to participate in this study. Pancreatic cancer was confirmed by reviewing the histopathologic slides of all patients both at Mansoura University and at M.D. Anderson Cancer Center. No patients with chronic pancreatitis were included in this study. Histopathologic examination of the tissues of the pancreatic cancer patients included in the study revealed no signs of chronic pancreatitis. All patients were Egyptian citizens, permanent residents of Dakahlia Province, and recruited before receiving chemotherapy or radiotherapy. There were no restrictions based on age, sex, or tumor stage. In addition, 52 comparison subjects were recruited from the earnose-throat and ophthalmology departments at Mansoura University General Hospital on the same medical campus of Mansoura University. The comparison subjects were chosen by systematic random sampling from inpatients admitted for surgeries related to nonchronic illnesses. After the nature of the study had been fully explained, informed oral and/or written consent was obtained from each person in the study.We used an interviewer-administered questionnaire, which included questions about lifetime occupational, residential, and smoking histories. Information was also collected about family history of pancreatic cancer.

### Sample collection.

For each patient and comparison subject, 10 mL of blood was collected into a sterile nonheparinized Vacutainer tube. The sample was allowed to clot for 5–10 min and then centrifuged for 10 min at 13,000 rpm. At least 2 mL of clear serum from each sample was transferred to glass tubes, which were labeled with patient name and identification number, clinic name, and collection time and date. Specimens were frozen at –20°C until they were transferred (within 3 weeks) to M.D. Anderson Cancer Center. Samples were hand-carried in dry ice during transport from Egypt to Houston to New Orleans, Louisiana, where they were stored at –80°C. The study was approved by the human subject committees of the Universities of Texas, Tulane, and Michigan in the United States and Mansoura University in Egypt.

### Modification of the one-step immunoassay for cadmium in serum.

**Figure 1:**
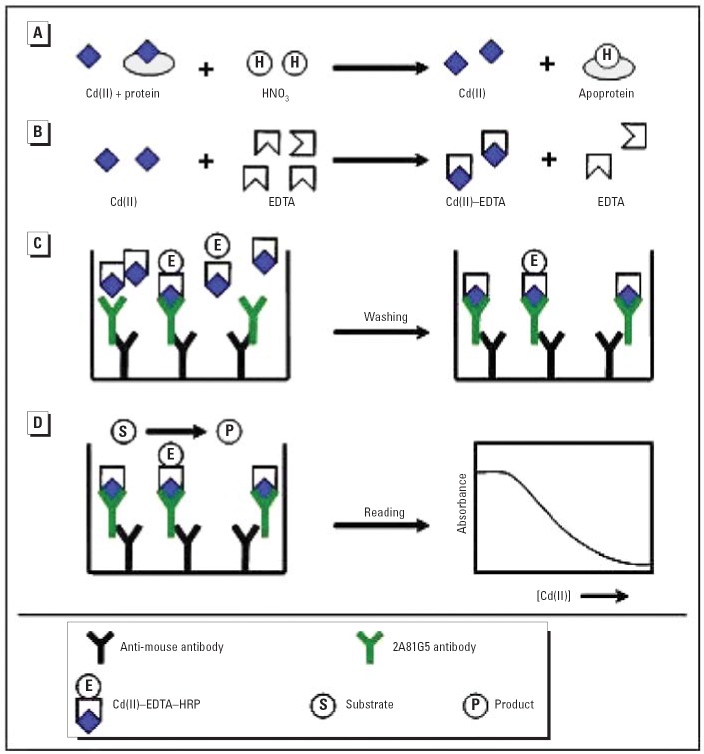
Modified one-step assay for Cd(II) in human serum. (*A*) Cadmium was first displaced from metallothionein and other proteins under acidic conditions. (*B*) An EDTA-containing buffer was added to the serum to form Cd(II)–EDTA complexes, which are recognized by the 2A81G5 antibody. (*C*) Microwell plates were coated and blocked as described in “Materials and Methods.” A serum sample was mixed 1:1 with enzyme-labeled Cd(II)–EDTA and subsequently added to the coated wells. The enzyme-labeled Cd(II)–EDTA competes with the Cd(II)–EDTA complexes from the serum sample for immobilized antibody binding sites. (*D*) Peroxidase substrate was added to the wells and absorbance was read at 450–650 nm.

The method described by Soliman et al. (2002) required relatively high quantities of the anti-cadmium monoclonal antibody, 2A81G5. Therefore, we modified the original method by using a goat anti-mouse antibody to capture and concentrate the 2A81G5 antibody on the microwell plate, as shown in Figure 1. Preliminary experiments demonstrated that when goat anti-mouse IgG, coated at a concentration of 2.0 μg/mL, was used to capture mouse monoclonal antibody, 5-fold less 2A81G5 was required for the subsequent cadmium assay (data not shown).

### Pooled human serum versus pooled Egyptian comparison serum.

We calculated standard curves in two separate sample matrices using the modified one-step immunoassay for cadmium. We compared a pooled human serum sample from Intergen Co. (Norcross, GA, USA) with a mixture of all the comparison samples from Egypt in a competition assay. Twenty-five microliters of each of the 52 Egyptian comparison samples were combined together and mixed well. Various concentrations of atomic absorption grade cadmium were spiked into the Egyptian pooled serum as well as Intergen’s pooled serum (Norcross, GA, USA). We then performed an immunoassay and compared inhibition curves.

### Determination of cadmium in human serum samples.

We diluted goat anti-mouse IgG_1_ (heavy chain specific; Jackson Immuno-Research Laboratories, West Grove, PA, USA) into HEPES-buffered saline (HBS; 137 mM NaCl, 3 mM KCl, and 10 mM HEPES, pH 7.4) to a concentration of 2 μg/mL. The diluted antibody (50 μL) was introduced to each well of a 96-well high-binding micro-well plate and incubated for 2 hr at 37°C. The plates were washed three times with phosphate-buffered saline containing 0.05% Tween-20. The wells were blocked with 3% bovine serum albumin in HBS at 37°C for 1 hr, followed by a wash step. The anti-Cd(II)–EDTA antibody, 2A81G5, was diluted to 0.5 μg/mL in HBS. A 50 μL aliquot of the diluted antibody was added to each well and incubated at room temperature for 1 hr, followed by a wash step.

Egyptian serum samples were thawed at room temperature and mixed well. A 75 μL aliquot of serum was added to 75 μL of 5% HNO_3_ and mixed well. The mixture was incubated at room temperature for 5 min and then spun at 15,000 rpm for 5 min. Acidification of the sample allows the release of cadmium from metallothionein and other protein ligands. A 90 μL aliquot of the supernatant was removed and conditioned with 10 μL of a 10X concentrated buffer (1.37 M NaCl, 30 mM KCl, 50 mM EDTA, 100 mM HEPES). The presence of EDTA allows the formation of Cd(II)–EDTA complexes. The sample was neutralized with 10 M KOH. Neutralized serum was added to an equal volume of 0.1 μg/mL Cd(II)–EDTA–HRP and mixed well. The mixture (50 μL/well) was added to three separate wells. After 1 hr incubation at room temperature, the plate was washed. 3,3′,5,5′-Tetramethylbenzidine peroxidase substrate (50 μL/well; Kirkegaard-Perry Laboratories, Gaithersburg, MD, USA) was added to each well, and color formation was stopped after 10 min with an equal volume of 1 N HCl. The absorbance of each well was measured in a dual-wavelength mode (450–650 nm) using a V_max_ Kinetic Microplate Reader (Molecular Devices Corporation, Sunnyvale, CA, USA). For preparation of a standard curve, pooled human serum from Intergen was spiked with various concentrations of atomic-absorption–grade cadmium standard and treated in a manner identical to that used for the serum samples noted above.

### Data analysis for the immunoassay.

**Figure 2:**
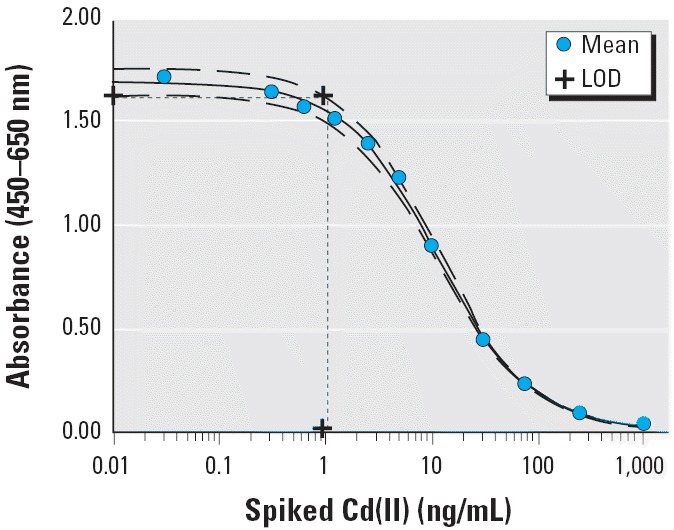
Typical standard curve using the one-step assay for Cd(II) in pooled human serum. See “Materials and Methods” for details. Data shown were the means of four replicates with a best-fit line (solid). Dashed lines on either side of the main curve represent the best-fit line of the mean values ± 2 SD.

We calculated standard curves on each immunoassay plate in replicates of four. We determined the mean value and SD of the absorbance provided by each cadmium concentration. These mean absorbance values (*y*) were plotted on a curve versus the cadmium concentrations (*x*) and used to fit the equation *y* = *a*_0_ – (*a*_1_ × *x*)/(*a*_2_ + *x*), where *y* is the experimental absorbance, *x* is cadmium concentration in the standard or sample, *a*_0_ is the absorbance in the absence of cadmium, *a*_1_ is the difference between the absorbance in the absence of cadmium and at a saturating concentration of cadmium, and *a*_2_ is the Cd(II) concentration that produces a 50% inhibition of signal (IC_50_). To account for the variability in the data, confidence bounds for each standard Cd(II) concentration were computed. The upper and lower curves were fit from the mean values ± 2 SD, respectively. The limit of detection (LOD) was determined with the use of the standard curves. The absorbance value for zero cadmium (*y*-intercept) for the lower curve (mean – 2 SD) was determined and then applied to the upper curve (mean + 2 SD) to find the cadmium concentration designated as the LOD. A visual depiction of how the LOD was determined is provided in Figure 2.

Each Egyptian serum sample was analyzed in triplicate in a particular assay. The mean absorbance value and SD were determined for each sample. We used SDs to help eliminate nonprecise values. Mean absorbance values for each serum sample were applied to the mean standard curve performed on the same plate to determine the cadmium concentration for that sample. When the sample was analyzed multiple times, the cadmium concentrations were averaged. Each original serum sample was diluted by a factor of 4.57 during the immunoassay procedure. To obtain the actual cadmium concentration of the serum samples, the assay cadmium concentration was multiplied by this number.

### Statistical methods.

For those samples that fell below the LOD (18 of 52), we used the method described by Helsel (1990) to assign a value for the cadmium concentration. In this method, one assumes that the actual cadmium concentration in these samples is described by a Gaussian distribution whose lower bound is zero and whose upper bound is the LOD of the immunoassay. The median cadmium concentration in these samples would be 50% of the LOD, and this value was therefore assigned to each nondetect sample. This is a conservative method of handling immunoassay data; the actual differences between the cases and the comparisons might be even greater than reported herein. The logarithm of serum cadmium level was used as the continuous exposure because the distribution of serum cadmium level was skewed. We used the Student’s *t*-test to test the differences among means of the logarithms of serum cadmium level. Because both transformed and untransformed analytical methods yielded comparable conclusions, we present the analysis based on the untransformed data for better comparison with other studies.

We also aimed to find a subset of predictors for pancreatic cancer disease status. The main response variable was disease status and potential predictors included age, sex, serum cadmium level, smoking, and farming. We analyzed the longest occupation reported by study subjects as farming and nonfarming. Farming jobs were defined as being a farmer or a housewife who lived in a rural area. Nonfarming occupations included administrators, barbers, businessmen, teachers, students, and women who lived in urban areas but reported their job title as “housewife.” Nonfarming also included industrial workers, such as painters and welders. We did not include residence in the final model because of its high correlation with occupation. Residence alone was not significant in a logistic regression model that did not include occupation. In this analysis, a study subject was considered a smoker if he or she reported ever smoking 100 cigarettes.

Multiple logistic regression models were fitted based on the variables chosen from the univariate analysis. The logistic regression models were designed to estimate the probability of having pancreatic cancer, whereas incorporating the covariates of age, sex, serum cadmium levels, occupation, and smoking. *p*-Values < 0.05 are reported as significant. There is no collinearity among the predictors. The Hosmer and Lemeshow goodness-of-fit test was used to test the model fitting. All the statistical tests were two sided. Data were analyzed using the Statistical Analysis System package (version 8.2; SAS Institute Inc., Cary, NC, USA).

## Results

### One-step immunoassay for cadmium.

In this study we used a previously published immunoassay procedure to quantify cadmium in serum samples. The assay was selective for cadmium; other metal ions, including manganese, cobalt, copper, zinc, magnesium, mercury, calcium, nickel, iron, and lead, did not significantly interfere with the assay even when tested at concentrations considerably higher than those present in human serum (Darwish and Blake 2002). The original one-step immunoassay for cadmium designed by Darwish and Blake (2002) required 125 ng of 2A81G5 antibody to be coated in each micro-well. With the large number of Egyptian serum samples to be assayed, an unacceptable quantity of antibody would have been required for these experiments. We therefore developed a modification of the original method that reduced the amount of 2A81G5 necessary for the capture of the Cd(II)–EDTA complexes by 5-fold. An overall schematic of the modified one-step assay is shown in Figure 1. In human serum, cadmium usually exists as a complex with metallothionein and other proteins. In order to determine the total level of cadmium in a serum sample, cadmium must be extracted from these proteins, a process most effectively achieved by acidification of the sample. Addition of a conditioning buffer containing EDTA allowed the formation of Cd(II)–EDTA complexes. This unlabeled Cd(II)–EDTA competed with a known concentration of enzyme-labeled Cd(II)–EDTA to bind to the 2A81G5 antibody immobilized on the microwell plate. After a wash step to remove unbound Cd(II)–EDTA and Cd(II)–EDTA–HRP, a chromogenic peroxidase substrate was added and HRP activity was detected. A standard curve was prepared by adding varying concentrations of atomic absorption grade cadmium to a pooled human serum sample that was treated in an identical manner as the patient samples. This standard curve was then used to determine the concentration of cadmium in each sample based on the observed spectrophotometric absorbance.

### Pooled human serum used preparation of the standard curve.

**Figure 3:**
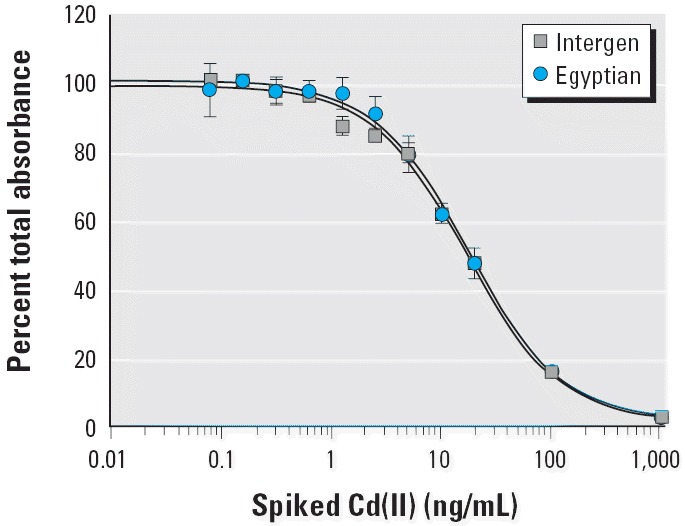
Pooled human serum versus pooled Egyptian comparison serum. Binding curves were calculated with cadmium spiked into the pooled human serum from Intergen or into a sample of pooled Egyptian comparison serum. IC_50_ values were very similar, 16.74 ng/mL and 17.44 ng/mL for Intergen and Egyptian samples, respectively. Values reported are mean ± SD (*n* ≥ 6).

In previous studies (Darwish and Blake 2002), a pooled human serum sample obtained from Intergen was used as the sample medium for preparing the standard curves on each of the immunoassay plates. In the present study, however, we were concerned that this pooled serum sample, from U.S. donors, might not be an appropriate medium for analysis of Egyptian donors. A large pooled serum sample from Egyptian donors was not available, so we prepared a small pooled sample by combining an equal volume (25 μL) of all the Egyptian noncancer comparison samples available from this study (a total of 52). The two pooled serum samples (from Intergen and the Egyptian comparisons) were spiked with various concentrations of cadmium and the subsequent inhibition curves were compared. As shown in Figure 3, the two curves were nearly identical. IC_50_ values were very similar, 16.74 ng/mL and 17.44 ng/mL for Intergen and Egyptian samples, respectively. These data indicate that the Egyptian samples did not contain any endogenous components that might affect immunoassay performance and that the pooled human serum from Intergen was an appropriate medium for all subsequent standard curves.

### Standard curve and determination of LODs in the immunoassay.

A typical standard curve, obtained by adding known concentrations of cadmium and EDTA to a pooled human serum sample, is shown in Figure 2. As the concentration of unlabeled cadmium increased, less of the enzyme-labeled Cd(II)–EDTA was able to bind to the 2A81G5 immobilized in the microwell; absorbance in the assay decreased as the unlabeled cadmium concentration increased. Four replicates of each standard concentration were analyzed. The average values of each four standard replicates were plotted on the graph and fitted using the equation described in “Materials and Methods” (Figure 2). Variability (2 × SD) was determined and graphed as described in “Materials and Methods” (Figure 2). These upper and lower limit curves were used to determine the LOD of the immunoassay, as shown in Figure 2. The LODs were determined for each standard curve and ranged between 0.4 and 1.0 ng/mL cadmium for the pancreatic cancer patients and between 0.3 and 1.0 ng/mL cadmium for the comparison subjects. These ranges show the consistency of standard curves performed from day to day and the high sensitivity of the assay. Each sample was assayed at least one time in triplicate. Many of the samples were assayed on multiple days, also in triplicate. The average absorbance value was detected and then applied to the mean standard curve like the one shown in Figure 2 to obtain a serum cadmium concentration.

### Characteristics of the study population.

There were no significant differences between the pancreatic cancer patients and the comparison subjects in terms of age, sex, residence, smoking status, or occupation, as shown in Table 1. Across the study population, age ranged from 21 to 75 years, with a median age of 52 in the cases and 49 in the comparison subjects. There were slightly more males than females in each group. Most study subjects (52% and 71% in cases and comparison subjects, respectively) were urban dwellers, and almost two-thirds were nonsmokers. Less than half of the cases had professional backgrounds, compared with two-thirds of the comparison subjects. There was, however, a significant difference in mean serum cadmium levels between the cases and comparisons (*p* = 0.012). In patients with pancreatic cancer, the mean cadmium level was 11.1 ± 7.7 ng/mL, whereas in the comparisons the mean ± SD was 7.1 ± 5.0 ng/mL. Although pancreatic cancer patients who reported histories of smoking showed higher but not significant differences in serum cadmium levels than those of comparison subjects who reported histories of smoking (11.8 ± 9.3 ng/mL and 7.1 ± 4.7 ng/mL, *p* = 0.14, in patients and comparison subjects, respectively), there were statistically higher serum cadmium levels in nonsmoker patients than in non-smoker comparison subjects (10.7 ± 6.9 ng/mL and 7.1 ± 5.3 ng/mL, *p* = 0.038, in patients and comparisons, respectively.

**Figure 4:**
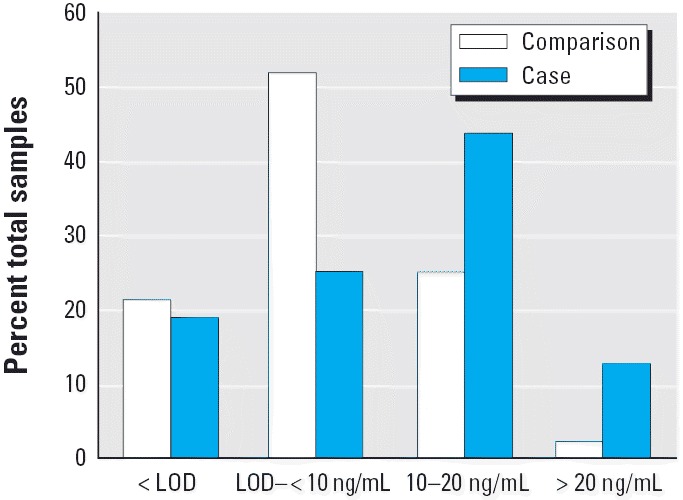
Distribution of samples and serum Cd(II) concentrations in comparison subjects versus pancreatic cancer patients. The values reported are the percentage of the total samples for that population.

A comparison of the different ranges of cadmium levels determined in the serum samples of the case and comparison populations is shown in Figure 4. Most comparison subjects exhibited serum cadmium levels between the LOD and < 10 ng/mL, with only one > 20 ng/mL Cd(II). The pancreatic cancer patients showed an overall trend toward higher serum cadmium concentrations. Most cases demonstrated serum cadmium levels between 10 and 20 ng/mL, and 4 of 31 exhibited cadmium levels > 20 ng/mL.

### Variation in cadmium levels in the study population.

Within the study population as a whole, there were no significant differences in serum cadmium levels as a function of sex, residence (rural or urban), or smoking status, as shown in Table 2. There was a trend toward significance on the basis of occupation (*p* = 0.0827) with farmers showing the highest levels (mean ± SD, 13.34 ± 8.29 ng/mL), followed by industrial workers (8.68 ± 5.46 ng/mL) and professionals (7.84 ± 6.14 ng/mL).

### Risk factors for pancreatic cancer.

The association between pancreatic cancer risk and subject age, residence (urban or rural), smoking status, and occupation (farming or non-farming) is indicated by the odds ratios (ORs) shown in Table 3. Both serum cadmium levels and farming were independently associated with increased risk of pancreatic cancer. The OR for serum cadmium levels was 1.12 [95% confidence interval (CI), 1.04–1.23; *p* = 0.0089]. Farming was associated with increased risk for pancreatic cancer OR = 3.25 (95% CI, 1.03–11.64) (Table 3).

## Discussion

In this pilot study, we tested the hypothesis that pancreatic cancer patients in this Egyptian population were exposed to higher levels of cadmium than were noncancer subjects. Serum cadmium levels were used as a marker for cadmium exposure. It is difficult to find a perfect dose estimator for cadmium. Urinary cadmium levels are often used; however, studies with animals and humans have shown that renal damage may lead to higher than normal cadmium excretion (Friberg et al. 1985; Nordberg and Piscator 1972). Jarup et al. (1997) have shown that blood cadmium can provide a better dose estimate than urinary cadmium concentrations, especially when tubular proteinuria is present. During high cadmium exposures, the cadmium in the blood increases relatively rapidly until, after some months, it reaches a concentration that corresponds to the intensity of exposure. If the exposure stops, the blood cadmium decreases with an initial half-time of 2–3 months (Elinder et al 1994; Jarup et al. 1983). Cadmium accumulated in the body, however, will continue to influence blood levels. Even after exposure ceases, the concentration in the blood never returns to preexposure levels. Thus blood cadmium has been proposed as one of the more accurate estimators of accumulated body burden (Alfven et al. 2002). Only about 10% of whole-blood cadmium is circulating in serum, but serum levels appear to correlate with blood levels (Lauwerys et al. 1994). The use of serum rather than whole blood in this study allowed us much greater flexibility in collecting, storing, and transporting patient samples.

In the patient population studied here, serum cadmium levels and farming were strong independent risk factors for pancreatic cancer, whereas age, sex, smoking, and residence were not. The highly significant association of pancreatic cancer with serum cadmium level is also consistent with other epidemiologic evidence. The strongest suspicion of an association between cadmium exposure and pancreatic cancer has been reported in Louisiana (Blot et al. 1978; Lemus et al. 1996; Tchounwou et al. 1996). Industrial activity along the Mississippi River has led to an accumulation of contaminants in southern Louisiana (Mielke et al. 2000). Seafood and rice are popular food items in the local diet. Both rice and fish harvested from cadmium-polluted areas may contain high levels of cadmium (Ikeda 1992; Modigosky et al. 1991). A case–control study in Louisiana showed a significantly increased risk for pancreatic cancer associated with rice consumption among Cajuns, with a dose–response relationship (Falk et al. 1988). Louisiana residents are also exposed to cadmium by inhalation. Lemus et al. (1996) have shown that 64 of 315 samples (20.3%) of indoor and outdoor air from 53 households in Louisiana exceeded the U.S. Environmental Protection Agency’s permissible levels for cadmium (Lemus et al. 1996). It is interesting to note the association between pancreatic cancer risk and farming in this study (OR = 3.25, *p* = 0.0475).

Although smoking has been identified as a strong risk factor for pancreatic cancer in previous studies (Doll et al. 1994; Howe et al. 1991), it was not significant in this study. There are at least two possible explanations for this finding. First, most subjects (approximately two-thirds) in both the case and comparison groups were nonsmokers. Second, cadmium is a by-product of cigarette smoke, and adjusting for serum cadmium levels in this study may have controlled for the effect of smoking. There was also no significant association between risk of pancreatic cancer and residence. Although rural residents might be expected to show increased risk because of exposure to pesticides and fertilizers (Anderson et al. 1996; Fontham and Correa 1989), exposure of urban industrial workers employed in manufacturing (Norell et al. 1986; Sheffet et al. 1982), metalworking (Maruchi et al. 1979; Rotimi et al. 1993; Silverstein et al. 1988), and soldering (Ji et al. 1999) may be equally significant. There may also be other important differences in cadmium exposure (e.g., diet and air quality) that distinguish urban from rural populations. Reports from urban areas in Egypt have shown high levels of cadmium in organ meats that are frequently consumed by the local population (Abou-Arab and Abou Donia 2000). A recent study on markers of environmental pollution in Egypt has also shown significantly higher levels of oxidative damage in urban populations, compared with rural populations (Soliman et al. 2004). The higher risk of pancreatic cancer associated with farming in this study may reflect the intense exposure to cadmium and other farming-related occupations in this population. Thus, two major risk factors may have overshadowed the relationship between smoking and cadmium levels in this study. The first was the exceptionally high levels of environmental pollution in our study region. The second included the high dietary intake of rice and fish grown in polluted soils and water of the study region. The National Food Consumption Study in Egypt has shown that residents of our study region of Dakahlia Province consume more fish and rice than does any other group in Egypt (Galal and Khorshid 1995). Unfortunately, neither detailed environmental exposures nor dietary intake was measured in the present study.

Studies with rodents showed the ability of the pancreas to accumulate high concentrations of cadmium. Mice injected with 4 mg/kg CdCl_2_ expressed a 2.5-fold increase in DNA synthesis in the pancreas, which Andrews et al. (1990) and Hellman (1986) interpreted as a sign of increased metallothionein synthesis. Rats injected with 4 and 8 mg/kg cadmium demonstrated 9.8- and 17.9-fold increases, respectively, in pancreatic metallothionein levels (Wormser and Calp 1988). Cadmium interferes with the use of essential metals such as calcium, zinc, selenium, and iron. Deficiencies of these essential metals, in conjunction with protein and vitamin deficiencies, exaggerate cadmium toxicity by increasing absorption through the gut and enhancing retention in different organs (Waalkes 1986). Studies on adult Wistar rats showed that zinc deficiency markedly increases cadmium accumulation in various organs (Waalkes 1986). Studies from our research region (Galal and Khorshid 1995) showed that 46% of women have a calcium intake < 50% of the required daily allowances (RDA); the consumption of B_12_, a marker of protein intake, is also < 50% of the RDA in 33% of women in the region. Nutritional deficiencies in our study region may be a major factor in cadmium accumulation in body organs of the local population.

Cadmium is a potent carcinogen in rodents. Injecting rats with CdCl_2_ resulted in a significant increase (from 2.2% to 8.5%) in the incidence of pancreatic islet cell tumors (Poirier et al. 1983). In experiments designed to determine the carcinogenic effects of repeated exposure to CdCl_2_, Fischer and Wistar rats showed a very high incidence of dose-related pancreatic metaplasia, as reflected by pancreatic hepatocyte formation (Konishi et al. 1990). These pancreatic hepatocytes are thought to arise from the ductal and interstitial cells of the pancreas, which resemble oval cells of the liver (Rao et al. 1986). Cadmium may activate oncogenes such as c-*myc*, *mdm2*, and cellular tumor antigen *p53*; inhibit tumor suppressor genes such as wild-type *p53* and *p27*; and consequently promote the proliferation of initiated cells (Fang et al. 2002).

The present study has several important strengths. The Dakahlia region has documented high levels of pollution and is therefore a unique setting for studying environmental risk factors and cancer occurrence. The stability of population without migration in Dakahliam maximizes the chances of life-long environmental exposure (Aly and Shields 1996). The high incidence of young pancreatic cancer patients (younger than 50 years of age) provides a unique model system for studying the effect of early environmental exposures on pancreatic carcinogenesis (Soliman et al. 2002). Finally, the wide range of occupational and lifestyle factors in this region provide help to investigate the occupational and epidemiologic risk factors associated with pancreatic cancer. The relatively small sample size of this rare cancer may limit the generalization of results.

In summary, this pilot study has shown a statistically significant association between pancreatic cancer and serum cadmium levels and farming. Future studies should expand on this pilot investigation by studying a larger number of pancreatic cancer patients and by collecting extensive information on the lifetime occupational, residential, and environmental exposures and dietary influences in order to clarify the role of cadmium in pancreatic cancer etiology in this population. Several molecular mechanisms have been identified by which cadmium may influence pancreatic cells (Chai et al. 1999; Merali and Singhal 1976; Shimoda et al 2001; Waalkes et al. 1992), and a better understanding of these processes is under study in our laboratories. It will also be important to examine genetic susceptibility and markers of genetic damage caused by environmental exposures to clarify the role of such exposures in pancreatic carcinogenesis.

## Figures and Tables

**Table 1:** Characteristics of the study population [*n* (%)].

Characteristic	Case (*n* = 31)	Comparison (*n* = 52)	*p*-Value
Age (years)			
< 60	21 (67.7)	41 (78.8)	0.3023
≥ 60	10 (32.3)	11 (21.2)	
Sex			
Male	19 (61.3)	30 (57.7)	
Female	12 (38.7)	22 (42.3)	0.8198
Residence			
Rural	15 (48.4)	15 (28.8)	0.0989
Urban	16 (51.6)	37 (71.2)	
Smoking			
Yes	11 (35.5)	21 (40.4)	
No	20 (64.5)	31 (59.6)	0.8161
Occupation			
Farmer	7 (22.6)	3 (5.8)	0.0805
Housewife	10 (32.3)	19 (36.5)	
Industrial	8 (25.8)	11 (21.2)	
Professional	6 (19.4)	19 (36.5)	
Farming-related occupation			
Yes	11 (35.5)	8 (15.4)	
No	20 (64.5)	44 (84.6)	0.0570
Serum cadmium level (ng/mL)			
Total			
Mean ± SD	11.1 ± 7.7	7.1 ± 5.0	0.0120
Median (range)	11.0 (27.9)	5.7 (24.2)	
Smokers			
Mean ± SD	11.8 ± 9.3	7.1 ± 4.7	0.1386
Median (range)	13.7 (27.0)	6.4 (16.5)	
Nonsmokers			
Mean ± SD	10.7 ± 6.9	7.1 ± 5.3	0.0380
Median (range)	11.0 (27.0)	5.5 (24.2)	

*p*-Values for quantitative variables were calculated using Student’s *t*-test; *p*-values for proportions and qualitative variables were calculated using the chi-square test.

**Table 2:** Serum cadmium concentration (ng/mL) in the combined patients and comparison sample.

	No.	Mean ± SD	Median (range)	*p*-Value
Sex				
Male	49	9.21 ± 7.09	7.77 (27.88)	
Female	34	7.72 ± 5.21	6.40 (19.19)	0.2966
Residence				
Rural	30	8.52 ± 6.31	7.31 (20.11)	0.9281
Urban	53	8.65 ± 6.51	6.86 (27.42)	
Smoking				
Yes	32	8.75 ± 6.86	7.08 (27.42)	
No	51	8.5 ± 6.16	6.86 (26.96)	0.8634
Occupation				
Farmer	10	13.34 ± 8.29	11.88 (1.83–28.79)	0.0827
Housewife	29	7.56 ± 6.05	5.94 (0.91–27.88)	
Industrial	19	8.68 ± 5.46	7.77 (0.91–16.45)	
Professional	25	7.84 ± 6.14	5.03 (1.37–25.14)	

*p*-Values for quantitative variables were calculated using Student’s *t*-test; *p*-values for proportions and qualitative variables were calculated using the chi-square test.

**Table 3:** ORs of risk factors for pancreatic cancer.

	Case *n* = 31)	Comparison *n* = 52)	OR (95% CI)	*p*-Value
Serum cadmium level (ng/mL, mean ± SD)	11.1 ± 7.7	7.1 ± 5.0	1.12 (1.04–1.23)	0.0089
Age [years, *n* (%)]				
< 60	21 (67.7)	41 (78.8)	1	0.1677
≥ 60	10 (32.3)	11 (21.2)	2.25 (0.71–7.26)	
Sex [*n* (%)]				
Male	19 (61.3)	30 (57.7)	2.06 (0.51–8.61)	0.311
Female	12 (38.7)	22 (42.3)	1	
Smoking [*n* (%)]				
Yes	11 (35.5)	21 (40.4)	0.54 (0.13–2.19)	0.3859
No	20 (64.5)	31 (59.6)	1	
Farming-related occupation [*n* (%)]				
Yes	11 (35.5)	8 (15.4)	3.25 (1.03–11.64)	0.0475
No	20 (64.5)	44 (84.6)	1	

ORs were calculated using logistic regression analysis. *p*-Values for quantitative variables were calculated using Student’s *t*-test; *p*-values for proportions and qualitative variables were calculated using the chi-square test.
